# Antimicrobial stewardship support for regional hospitals in Japan: common challenges and practical strategies for implementation including the need for implementation science

**DOI:** 10.1017/ash.2025.10188

**Published:** 2025-10-08

**Authors:** Yasuaki Tagashira, Yoshiaki Gu

**Affiliations:** 1 Department of Infectious Diseases, Institute of Science Tokyo Graduate School of Medical and Dental Sciences, Tokyo, Japan; 2 Center for Infectious Disease Education and Analysis (TCIDEA), Institute of Science Tokyo, Tokyo, Japan

## Context

Antimicrobial stewardship programs (ASPs) are being promoted globally as direct and effective means of addressing the serious threat of antimicrobial resistance (AMR).^1^ However, most Japanese community hospitals lack full-time infectious disease (ID) specialists to lead ASPs. Indeed, the number of ID specialists is small in comparison to the large number of hospitals in Japan, thus forcing pharmacists, clinical laboratory technologists, nurses, and non-ID physicians to carry the burden of managing ASPs. The present report describes the support provided by an external ID specialist to 9 hospitals and highlights the intermediary role of the specialist in facilitating multidisciplinary collaboration and positioning a remote antimicrobial stewardship team (AST) support model as a capacity-building adjunct to extend leadership support to hospitals without on-site ID specialists.

## Which problem was the author aiming to address?

Hospitals with ID specialists have been successful in reducing the use of broad-spectrum antimicrobials by providing ID consultations and by directing ASPs.^2^ However, hospitals in Japan are rarely able to provide expert ID consultation owing to the shortage of qualified personnel. Moreover, although Japan’s National Action Plan on AMR financially incentivized the formation of AST, much less time is allocated to AST activities than in the West.^3^ Additionally, the implementation of evidence-based interventions often depends heavily on the initiative of individual team members. Thus, the present study aimed to assess circumstances at local hospitals and the latter’s need for targeted support.

## How was the problem solved?

A survey of ASP activities was first conducted at 9 hospitals. Later, an external ID specialist held online meetings to ascertain the issues faced by each facility. Supplementary Table 1 shows the characteristics of the participating hospitals. During discussions, the staff voiced concerns, such as “We don’t know how to implement an ASP effectively,” “We were active at first when the financial incentive was introduced but couldn’t sustain the effort,” and “Our approach is inconsistent.” Common issues included the absence of standard operating procedures and local guidelines, the failure to use available guidelines, and the lack of a clear decision-making process. In-person visits to the hospitals were then conducted to discuss these circumstances with the staff and tailor recommendations to the facility’s needs. Support included interventions which were previously proven to be effective in Japan, such as postprescription audit and feedback for broad-spectrum antimicrobials, diagnostic stewardship for *Clostridioides difficile* infection, selective reporting, and development of local guidelines for the treatment of common IDs.^2,4^ Because of differences in needs and the feasibility of various interventions across the hospitals, a uniform method was considered unrealistic. Instead, the support was tailored to the requirements of each facility, and continuous follow-up was provided. This external support also fulfilled the need for leadership in coordinating multidisciplinary teams.

## What scientific lessons were learned?

The proposed interventions were feasible and sustainable in hospitals with ID specialists, but in those without specialists, a simple, standard procedure manual alone often proved insufficient. Potential barriers to smooth implementation included discrepancies in the level of knowledge about ASP among the AST members; the reluctance of physicians not specializing in ID to respond to questions about ASP, which hampered the dissemination of important information; and the tendency for the non-physician members of the team to rely on the lead physician to initiate communication. Strengthening interdisciplinary information-sharing was critical to the AST’s efficacy, especially given the need to fill the knowledge gaps of the various members. Quantitative analysis of changes in antimicrobial use and patient outcomes was not feasible because of the hospitals’ differences in readiness and our inability to identify and address local barriers fully before making recommendations. These facts underscore the value of systematically identifying facilitators of, and barriers to, ASPs. Furthermore, short-term support alone was unlikely to achieve sustained improvement; engagement by ASP specialists increased both the continuity and scalability of the support. Therefore, an ongoing, remote AST support model designed to extend the leadership and coordination of antimicrobial stewardship across hospitals may be effective in Japan if the prerequisites stated above are met. The model is an adjunct, not a replacement, for trained, on-site personnel. Table 1 summarizes the actions, facts, barriers, and solutions.

**Table 1.** “What we did/what improved/what did not improve/major barriers/proposed solutions”

**What we did**
Assessed ASP activities across 9 hospitals. Held online meetings with each AST and conducted visits to individual sites. Identified barriers and made facility-specific recommendations. Supported implementation (eg, PAF, selective susceptibility reporting, *C. difficile*-related diagnostic stewardship, local treatment guidelines). Provided external leadership to coordinate multidisciplinary work and follow-up.
**What improved**
Coordination and leadership across teams. Continued engagement via expert support, enabling continuity and scale. Early wins boosting staff motivation and willingness to change behavior.
**What did not improve**
Uniform quantitative evaluation (eg, antibiotic use and outcomes) across sites. Implementation at sites without ID specialists when relying on written protocols alone. Sustainability with short-term support only. Persistent barriers: knowledge gaps among AST members; reluctance among non-ID physicians; reliance of non-physician staff on lead physicians; absent/underused local guidelines; unclear decision-making pathways.
**Major barriers**
Knowledge gaps among AST members Reluctance and lack of confidence among non-ID physicians Reliance of non-physician staff on lead physicians Lack of standardized consumption/outcome metrics
**Proposed solution**
Concise, case-based micro-learning and a Japanese-language online ASP course for scalable, self-paced training (already developed by the authors) A staged measurement plan: cross-site process indicators first, then DDD/DOT and action metrics (de-escalation, stop, escalation, IV-to-PO), followed by outcome linkage.

Note. ASP, antimicrobial stewardship program; AST, antimicrobial stewardship team; PAF, prospective audit and feedback; ID, infectious diseases; DDD, defined daily dose; DOT, days of therapy.

## What leadership or management lessons were learned?

The role of external support providers encompassed more than the provision of technical expertise; they also, critically, collaborated with local staff to identify and solve issues, especially in facilities without an ID specialist, to lead the ASP. Their success required a system that facilitated the staff’s decision-making and speedy access to consultation. Furthermore, achieving success incrementally served to motivate and encourage further behavioral changes. Effective ASP implementation ultimately relies on processes designed to bolster local ownership and promote shared understanding.Finally, a remote AST support modeled by ID specialists and a multidisciplinary team may contribute to successful ASP implementation as a capacity-building adjunct rather than a replacement for on-site personnel.
